# Author Correction: Multi-cohort and longitudinal Bayesian clustering study of stage and subtype in Alzheimer’s disease

**DOI:** 10.1038/s41467-024-50044-2

**Published:** 2024-07-10

**Authors:** Konstantinos Poulakis, Joana B. Pereira, J.-Sebastian Muehlboeck, Lars-Olof Wahlund, Örjan Smedby, Giovanni Volpe, Colin L. Masters, David Ames, Yoshiki Niimi, Takeshi Iwatsubo, Daniel Ferreira, Eric Westman, Japanese Alzheimer’s Disease Neuroimaging Initiative, Australian Imaging, Biomarkers and Lifestyle study

**Affiliations:** 1 Division of Clinical Geriatrics, Department of Neurobiology, Care Sciences and Society, Karolinska Institutet, Stockholm, Sweden; 2 Clinical Memory Research Unit, Department of Clinical Sciences, Lund University, Malmo, Sweden; 3 Department of Biomedical Engineering and Health Systems (MTH), KTH Royal Institute of Technology, Stockholm, Sweden; 4 Department of Physics, University of Gothenburg, Gothenburg, Sweden; 5 The Florey Institute of Neuroscience and Mental Health, The University of Melbourne, Victoria, Australia; 6 Academic Unit for Psychiatry of Old Age, St George’s Hospital, University of Melbourne, Melbourne, Victoria Australia; 7 National Ageing Research Institute, Parkville, Victoria Australia; 8 Unit for Early and Exploratory Clinical Development, The University of Tokyo Hospital, Tokyo, Japan; 9 Department of Radiology, Mayo Clinic, Rochester, MN USA; 10 Department of Neuroimaging, Centre for Neuroimaging Sciences, Institute of Psychiatry, Psychology and Neuroscience, King’s College London, London, UK

**Keywords:** Machine learning, Predictive markers, Alzheimer's disease, Alzheimer's disease

Correction to: *Nature Communications* 10.1038/s41467-022-32202-6, published online 5 August 2022

The original version of this Article contained an error in Tables 1, 3 and Supplementary Table S7. In Table 1, the correct version of the 2nd row of the 1st column in both the Discovery dataset and Validation dataset states ‘females’ instead of the original, incorrect ‘males’. In Table 3, the correct version of the 2nd row of the 1st column states ‘females’ instead of the original, incorrect ‘males’. In Supplementary Table S7 the correct version of the 2nd row of the 1st column under both the ADNI and J-ADNI/AIBL headings states ‘females’ instead of the original, incorrect ‘males’.

For Tables 1 and 3 this has been corrected in both the PDF and HTML versions of the Article. The HTML has been updated to include a corrected version of the Supplementary Information.

### Supplementary information

Updated Supplementary Information

