# Supplementary material for: Multi-cohort and longitudinal Bayesian clustering study of stage and subtype in Alzheimer’s disease
Source: Nat Commun. 2022 Aug 5;13:4566. doi: 10.1038/s41467-022-32202-6 (PMC9355993; doi:10.1038/s41467-022-32202-6)
Supplement: Supplementary file 1 — Supplementary Information [file 41467_2022_32202_MOESM1_ESM.pdf]

Multi-cohort and longitudinal Bayesian clustering study of stage and subtype in Alzheimer's disease

Appendix

Methods related notes

J-ADNI

Data used in preparation of this article were obtained from the Japanese Alzheimer's Disease Neuroimaging Initiative (J-ADNI) database deposited in the National Bioscience Database Center Human Database, Japan (Research ID: hum0043.v1, 2016). The J-ADNI was launched in 2007 as a public-private partnership, led by Principal Investigator Takeshi Iwatsubo, MD. The primary goal of J-ADNI has been to test whether serial magnetic resonance imaging (MRI), positron emission tomography (PET), other biological markers, and clinical and neuropsychological assessment can be combined to measure the progression of late mild cognitive impairment (MCI) and mild Alzheimer's disease (AD) in the Japanese population.

J-ADNI was supported by the following grants: Translational Research Promotion Project from the New Energy and Industrial Technology Development Organization of Japan; Research on Dementia, Health Labor Sciences Research Grant; Life Science Database Integration Project of Japan Science and Technology Agency; Research Association of Biotechnology (contributed by Astellas Pharma Inc., Bristol-Myers Squibb, Daiichi-Sankyo, Eisai, Eli Lilly and Company, Merck-Banyu, Mitsubishi Tanabe Pharma, Pfizer Inc., Shionogi & Co., Ltd., Sumitomo Dainippon, and Takeda Pharmaceutical Company), Japan, and a grant from an anonymous Foundation.

J-ADNI study group

Takeshi Iwatsubo, Takashi Asada, Hiroyuki Arai, Morihiro Sugishita, Hiroshi Matsuda, Fumio Yamashita, Kengo Ito, Michio Senda, Kenji Ishii, Ryozo Kuwano, Yasuo Ihara.

AIBL

The AIBL study was approved by the institutional ethics committees of Austin Health, St Vincent's Health, Hollywood Private Hospital and Edith Cowan University, and all volunteers gave written informed consent before participating in the study.

Core funding for the AIBL study was provided by CSIRO, which was supplemented by "in kind" contributions from the study partners (supplementary material, page 1). The AIBL investigators thank Richard Head of CSIRO for initiating and facilitating the AIBL collaboration. The study also received support from the National Health and Medical Research Council via the Dementia Collaborative Research Centre's program (DCRC2). Pfizer International has contributed financial support to assist with the analysis of blood samples and to further the AIBL research program. Ashley Bush is supported by a Federation Fellowship from the Australian Research Council. Cassandra Szoeke is partially supported by a research fellowship funded by Alzheimer's Australia. Alzheimer's Australia (Victoria and Western Australia) assisted with the promotion of the study and the screening of telephone calls

from volunteers. The AIBL team wishes to thank the following clinicians who referred patients with AD and/or MCI to the study: Professor David Ames, Associate Professor Brian Chambers, Professor Edmond Chiu, Dr Roger Clarnette, Associate Professor David Darby, Dr Mary Davison, Dr John Drago, Dr Peter Drysdale, Dr Jacqui Gilbert, Dr Kwang Lim, Professor Nicola Lautenschlager, Dr Dina LoGiudice, Dr Peter McCardle, Dr Steve McFarlane, Dr Alastair Mander, Dr John Merory, Professor Daniel O'Connor, Professor Christopher Rowe, Dr Ron Scholes, Dr Mathew Samuel, Dr Darshan Trivedi, and Associate Professor Michael Woodward. We thank all those who participated in the study for their commitment and dedication to helping advance research into the early detection and causation of AD.

AIBL study group

Oscar Acosta, David Ames, Jennifer Ames, Manoj Agarwal, Alex Bahar-Fuchs, David Baxendale, Kiara Bechta-Metti, Carlita Bevage, Lindsay Bevege, Pierrick Bourgeat, Belinda Brown, Ashley Bush, Roger Clarnette, Tiffany Cowie, Kathleen Crowley, Andrew Currie, David Darb,y Daniela De Fazio, Denise El-Sheikh, Kathryn Ellis, Kerryn Dickinson, Maree Farrow, Noel Faux, Jonathan Foster, Jurgen Fripp, Christopher Fowler, Veer Gupta, Peter Hudson, Gareth Jones, Jane Khoo, Asawari Killedar, Neil Killeen, Tae Wan Kim, Eleftheria Kotsopoulos, Rebecca Lachovitzki, Nicola Lautenschlager, Nat Lenzo, Qiao-Xin Li, Xiao Liang, Kathleen Lucas, James Lui, Georgia Martins, Ralph Martins, Paul Maruff, Colin Masters, Andrew Milner, Claire Montague, Lynette Moore, Audrey Muir, Christopher O'Halloran, Graeme O'Keefe ,Anita Panayiotou, Athena Paton, Jacqui Paton, Jeremiah Peiffer, Svetlana Pejoska, Kelly Pertile, Kerryn Pike, Lorien Porter, Roger Price, Parnesh Raniga, Glenn Rees, Alan Rembach, Miroslava Rimajova, Peter Robins, Elizabeth Ronsisvalle, Rebecca Rumble, Mark Rodrigues, Christopher Rowe, Olivier Salvado, Jack Sach, Mathew Samuel, Greg Savage, Gobhathai Sittironnarit, Cassandra Szoeke, Kevin Taddei Tania Taddei, Darshan Trivedi, Brett Trounson, Marinos Tsikkos, Victor Villemagne, Stacey Walker, Vanessa Ward, Michael Woodward, Olga Yastrubetskaya.

ADNI

The ADNI was launched in 2003 as a public-private partnership, led by a principal investigator Michael W. Weiner, MD. The primary goal of ADNI has been to test whether serial MRI, positron-emission tomography, other biological markers, and clinical and neuropsychological assessments can be combined to measure the progression of MCI and early AD. Similar to the AddNeuroMed study, ADNI strives to reveal sensitive and specific markers of AD progression in patients from various sites to support the development of new treatments and monitor their effectiveness, as well as to reduce the expenditures of clinical trials. Informed consent was obtained from all subjects included in this study. For AD patients, consent was obtained both from the patient and a relative.

AddNeuroMed

AddNeuroMed is an integrated project that is part of InnoMed (the Innovative Medicines Initiative) and funded by the European Union Sixth Framework program. The main objective of AddNeuroMed is to identify biomarkers or experimental models that can improve diagnosis, prediction, and monitoring of disease progression in AD. Regarding neuroimaging, AddNeuroMed uses MRI and magnetic resonance spectroscopy to extract valuable information for the identification of AD biomarkers (Westman et al., 2011). The MRI data for AddNeuroMed were collected from different centres across Europe: University of Perugia (Italy), King's College London (United Kingdom), Aristotle University of Thessaloniki (Greece), University of Kuopio (Finland), University of Lodz (Poland), and University of Toulouse (France). Informed consent was obtained from all subjects included in this study. For AD patients, consent was obtained both from the patient and a relative.

This AddNeurMed study was supported by InnoMed, (Innovative Medicines in Europe) an Integrated Project funded by the European Union of the Sixth Framework Programme priority FP6-2004-LIFESCIHEALTH-5, Life Sciences, Genomics and Biotechnology for Health.

	ADNI	JADNI	AIBL	AddNeuroMed	Visit Interval	1st quartile	Mean	Median	2nd quartile
					Discovery dataset				
1st	207	90	23	-					
					1st – 2nd visit	0·3	0·68	0·5	1
2nd	207	90	23	-					
					2nd–3rd visit	0·3	0·62	0·5	1
3rd	162	73	8	-					
					3rd-4th visit	0·3	0·7	0·5	1
4th	80	52	2	-					
					4th-5th visit	1	1	1	1
5th	23	5	1	-					
6th	1	0	0	-					
7th	1	0	0	-					
					Validation dataset				
1st	216	168	67	120					
					1st – 2nd visit	0·25	0·33	0·25	0·25
2nd	0	0	0	101					
					2nd–3rd visit	0·75	0·75	0·75	0·75
3rd	0	0	0	64					

Table S1. Longitudinal visit time intervals

The columns 2-5 show visit frequencies for each cohort (ADNI, JADNI, AIBL, AddNeuroMed) and dataset (Discovery, Validation). Columns 6-9 Show the time in years between pairs of visits (e.g. the mean time interval between the 1st and 2nd MRI visits of AD patients in the discovery cohort was 0.68 years).

Clustering solution	Cluster means with high autocorrelation, %	Cluster means with high autocorrelation, N	Model deviance, -2*log*(likelihood)**
2	27·2	17	19056
5	27·9	25	18656
4	28·3	19	18470
3	25·2	23	18898
6	29·8	26	18616
7	31·2	36	81263
8	22·7	60	67695

Table S2. Model optimisation information

Five models were optimized for each number of clusters between two and eight. This totalled thirty-five models. Initial values were altered for each different model simulation. The best model for each number of clusters is presented above. The 2-cluster solution has slightly lower percentage of means with highly autocorrelated Monte Carlo Markov chains followed by the 5-cluster solution (even those chains with higher autocorrelation compared to the ones with no autocorrelation have converged but not as optimally as the latter ones). Model deviance is slightly lower for the latter model. This shows that the 2 and 5-cluster solutions do not differ significantly in terms of model quality. **With the word likelihood of the model we refer to the observed data likelihood[1]. The order of clustering solution on the table was decided based on a formula that accounts cluster means with high autocorrelation (a), and model deviance (b), simultaneously. That is, a and b were rescaled between 0 and 1 in order to be equally weighted. Then Euclidean distance was calculated based on a and b values for each clustering solution. The solution with the smallest Euclidean value accounts for the most optimal clustering solution (lowest a and b) followed by the other solutions ordered in the same fashion.

	Minimal	Limbic predominant	Limbic predominant +	Diffuse	Hippocampal sparing	Minimal
**N, N(%)**	377 (56.3%)	238 (35.5%)	33 (4.9%)	12 (1.8%)	10 (1.5%)	377 (56.3%)
**PSYCH, N(%)**	100 (26.5%)	65 (27.3%)	9 (27.3%)	6 (50%)	2 (20%)	100 (26.5%)
**NEURL, N(%)**	69 (18.3%)	46 (19.3%)	4 (12.1%)	4 (33.3%)	1 (10%)	69 (18.3%)
**HEAD, N(%)**	167 (44.3%)	104 (43.7%)	16 (48.5%)	2 (16.7%)	5 (50%)	167 (44.3%)
**CARD, N(%)**	211 (56%)	141 (59.2%)	16 (48.5%)	4 (33.3%)	4 (40%)	211 (56%)
**RESP, N(%)**	64 (17%)	42 (17.6%)	1 (3%)	3 (25%)	1 (10%)	64 (17%)
**HEPAT, N(%)**	25 (6.6%)	9 (3.8%)	0 (0%)	1 (8.3%)	0 (0%)	25 (6.6%)
**DERM, N(%)**	74 (19.6%)	55 (23.1%)	6 (18.2%)	0 (0%)	0 (0%)	74 (19.6%)
**MUSCL, N(%)**	176 (46.7%)	109 (45.8%)	13 (39.4%)	4 (33.3%)	1 (10%)	176 (46.7%)
**ENDO, N(%)**	138 (36.6%)	93 (39.1%)	12 (36.4%)	6 (50%)	1 (10%)	138 (36.6%)
**GAST, N(%)**	124 (32.9%)	93 (39.1%)	6 (18.2%)	2 (16.7%)	0 (0%)	124 (32.9%)
**HEMA, N(%)**	13 (3.4%)	14 (5.9%)	1 (3%)	0 (0%)	0 (0%)	13 (3.4%)
**RENA, N(%)**	142 (37.7%)	74 (31.1%)	11 (33.3%)	2 (16.7%)	2 (20%)	142 (37.7%)
**ALLE, N(%)**	103 (27.3%)	65 (27.3%)	6 (18.2%)	1 (8.3%)	0 (0%)	103 (27.3%)
**ALCH, N(%)**	13 (3.4%)	7 (2.9%)	3 (9.1%)	0 (0%)	1 (10%)	13 (3.4%)
**SMOK, N(%)**	148 (39.3%)	83 (34.9%)	11 (33.3%)	6 (50%)	1 (10%)	148 (39.3%)
**MALI, N(%)**	75 (19.9%)	37 (15.5%)	4 (12.1%)	3 (25%)	2 (20%)	75 (19.9%)
**SURG, N(%)**	200 (53.1%)	125 (52.5%)	15 (45.5%)	8 (66.7%)	4 (40%)	200 (53.1%)

Table S3. Medical history summary.

The data presented here compile a medical history summary for the AD patients belonging in the five discovered clusters (discovery and validation datasets). They are only available for a subset of the ADNI and J-ADNI cohorts and thus are not reported as main finding in the study due to no representativity in the AIBL or AddNeuroMed cohorts. PSYCH (Psychiatric), NEURL (Neurologic other than AD), HEAD (Head, Eyes, Ears, Nose, and Throat), CARD (Cardiovascular), RESP (Respiratory), HEPAT (Hepatic), DERM (Dermatologic-Connective Tissue), MUSCL (Musculoskeletal), ENDO (Endocrine-Metabolic), GAST (Gastrointestinal), HEMA (Hematopoietic-Lymphatic), RENA (Renal-Genitourinary), ALLE (Allergies or Drug Sensitivities), ALCH (Alcohol Abuse), SMOK (Smoking), MALI (Malignancy), SURG (Major Surgical Procedures).

		Dementia duration at MRI		
		**<50 months**	**50-99 months**	>99 months
Discovery dataset	MA	408	199	1
	LPA	229	43	-
	LPA+	64	7	-
	DA	13	-	4
	HS	30	1	-
Validation dataset	MA	274	125	6
	LPA	216	54	-
	LPA+	12	-	-
	DA	13	-	-
	HS	8	-	-

Table S4. Dementia duration at MRI acquisition for each AD patient cluster.

The data in the table refer to MRI visits per cluster of patients in the different disease duration spans (<50 months, 50-99 months, and >99 months).

**Clustering solution**	**MA**	**LPA**
MA	-	2
LPA	16	-
LPA+	5	0
DA	0	0
HS	1	1

Table S5. First and second order subject assignment into clusters for the discovery dataset.

The clustering algorithm assigned probabilities of each subject to belong in each cluster. Information on 2nd and 3rd class assignment is presented for subjects that did not have high probability to belong in the 1st cluster assignment (e.g. 16 subjects that are predominantly clustered in the LPA cluster can also be clustered to the MA cluster with lower probability while only one subject of the HS cluster can be clustered to the MA cluster with a lower probability than for the HS cluster that is assigned to).

**Clustering solution**	**MA**	**LPA**	**HS**
MA	-	1	1
LPA	0	-	0
LPA+	1	1	0
DA	1	0	0
HS	0	0	-

Table S6. First and second order subject assignment into clusters for the validation dataset.

The classification algorithm (post clustering) assigned probabilities of each subject to belong in each cluster. Information on 1nd and 2rd class assignment is presented for subjects that did not have high probability to belong in the 1st cluster assignment. Only 5 out of the 571 AD patients did not receive a certain classification to any of the clusters (e.g. first row: one patient that IS predominantly clustered in the MA cluster can also be clustered to the LPA cluster with lower probability and one subject of the MA cluster can be clustered to the HS cluster with a lower probability.

			ADNI			
	Cluster 1	Cluster 2	Cluster 3	Cluster 4	Cluster 5	Statistics p[*1]
**N[1]**	26 (12.6%)	46 (22.2%)	131 (63.3%)	3 (1.4%)	1 (0.5%)	
**Females[1, e]**	14 (53.8%)	27 (58.7%)	48 (36.6%)	2 (66.7%)	-	<0.05[a]
**Cohort ADNI[1]**	26 (100%)	46 (100%)	131 (100%)	3 (100%)	1 (100%)	-
**Age[2, a]**	78.6 (5.3)	74 (8)	75.8 (6.8)	71.9 (11.6)	56.5	<0.05[b]
**AD onset Age[2]**	75 (7.4)	72 (8.9)	72 (7.4)	66 (7.4)	55	0.17[b]
**Education class[3]**	3.23 (0.82)	3.24 (0.85)	3.42 (0.83)	3.67 (0.58)	4	0.38[a]
**Apoe e4[1,4]**	18 (69.2%)	31 (67.4%)	103 (78.6%)	2 (66.7%)	1 (100%)	0.12[a]
**Apoe e2[1,4]**	2 (7.7%)	2 (4.3%)	4 (3.1%)	-	-	0.53[a]
**MMSE[3]**	23 (2.2)	22.71 (2.2575)	23.32 (2.0)	23.33 (2.3)	21	0.19[b]
**CDR[3]**	0.86 (0.2)	0.79 (0.2)	0.77 (0.2)	0.75 (0.3)	1	0.35[a]
**CDR sum of boxes[3]**	4.86 (1.6)	4.83 (1.6)	4.34 (1.4)	5.25 (2.4)	4.5	0.18[b]
			**J-ADNI/AIBL**			
	Cluster 1	Cluster 2	Cluster 3	Cluster 4		
**N[1]**	2 (1.8%)	46 (40.7%)	63 (55.8%)	2 (1.8%)		
**Females[1]**	1 (50%)	26 (56.5%)	37 (58.7%)	1 (50%)		0.97[a]
**Cohort AIBL**	2 (100%)	16 (34.8%)	4 (6.3%)	1 (50%)		-
**Cohort J-ADNI[11]**	-	30 (65.2%)	59 (93.7%)	1 (50%)		-
**Age[2]**	58.8 (5.3)	73.6 (8.4)	75.1 (7.6)	64.8 (4.9)		0.70[b]
**AD onset Age[2]**	57.7 (3.9)	73 (8.7)	72 (7.4)	64.2 (5.6)		0.84[b]
**Education class[3]**	2	2.65 (1.04)	2.54 (0.86)	2.5 (0.71)		0.18[a]
**Apoe e4[1,4]**	1 (50%)	31 (67.4%)	40 (63.5%)	-		0.99[a]
**Apoe e2[1,4]**	-	3 (6.5%)	1 (1.6%)	-		0.40[a]
**MMSE[3]**	19.5 (2.1)	23.06 (2.3)	22.61 (2.1)	16 (9.8)		0.34[b]
**CDR[3]**	0.75 (0.3)	0.65 (0.2)	0.64 (0.2)	0.75 (0.3)		0.83[a]

**CDR sum of boxes**[3]	4.75 (1.7)	3.53 (1.3)	3.66 (1.3)	5 (1.4)	0.67[b]

Table S7. Demographical and cognition, characteristics of the separate cohort analyses.

Notes: [1] n (%); [2] median (median absolute distance); [3] mean (sd); [4]the percentage denominator refers to the sum of the non-missing Apoe records; Education years are categorized in 4 classes (1 =< 0-8 years; 2 = 9-13 years; 3 = 13-15 years, 4 > 15 years); MMSE: Mini mental state examination; CDR: Clinical dementia rating: CDR: CDR sum of boxes; statistical testing: for ADNI only clusters 1, 2, and 3 were assessed because clusters 4 and 5 have low counts to test hypotheses. For J-ADNI,/AIBL only differences between clusters 2 and 3 were assessed because clusters 1 and 4 have low counts to test hypotheses; [a] categorical variables comparisons with χ^2 (two sided) and Fisher's exact test (two sided); [b] group comparisons with Kruskal-Wallis rank sum test (non-parametric); The Hochberg method was employed for post hoc multiple comparisons corrections. Confidence level for statistical comparisons was set to a = 0.05.

[c] Significant differences between ADNI clusters 1 and 2

[d] Significant differences between ADNI clusters 1 and 3

[e] Significant differences between ADNI clusters 2 and 3

		Combined cohort analysis clusters				
		**MA**	**LPA**	LPA+	**DA**	**HS**
ADNI	Cluster 1	15	0	10	0	1
	Cluster 2	3	36	25	0	0
	Cluster 3	101	25	3	0	2
	Cluster 4	0	0	0	3	0
	Cluster 5	0	0	0	0	1
J-ADNI and AIBL	Cluster 1	1	0	0	0	1
	Cluster 2	13	29	2	1	1
	Cluster 3	56	3	4	0	0
	Cluster 4	0	0	0	0	2

Table S8. Concordance between the clustering in the whole AD dataset analysis and the separate cohort analyses.

Cortical regions (thickness)	Subcortical regions (volume)
Banks superior temporal sulcus	Thalamus-Proper
Caudal anterior-cingulate cortex	Caudate
Caudal middle frontal gyrus	Putamen
Cuneus cortex	Pallidum
Entorhinal cortex	Hippocampus
Fusiform gyrus	Amygdala
Inferior parietal cortex	Accumbens-area
Inferior temporal gyrus	
Isthmus–cingulate cortex	
Lateral occipital cortex	
Lateral orbital frontal cortex	
Lingual gyrus	
Medial orbital frontal cortex	
Middle temporal gyrus	
Parahippocampal gyrus	
Paracentral lobule	
Pars opercularis	
Pars orbitalis	
Pars triangularis	
Pericalcarine cortex	
Postcentral gyrus	
Posterior-cingulate cortex	
Precentral gyrus	
Precuneus cortex	
Rostral anterior cingulate cortex	
Rostral middle frontal gyrus	
Superior frontal gyrus	
Superior parietal cortex	
Superior temporal gyrus	
Supramarginal gyrus	
Frontal pole	
Temporal pole	
Transverse temporal cortex	
Insula cortex	

Table S9. List of cortical and subcortical ROIs that were included in the analysis.

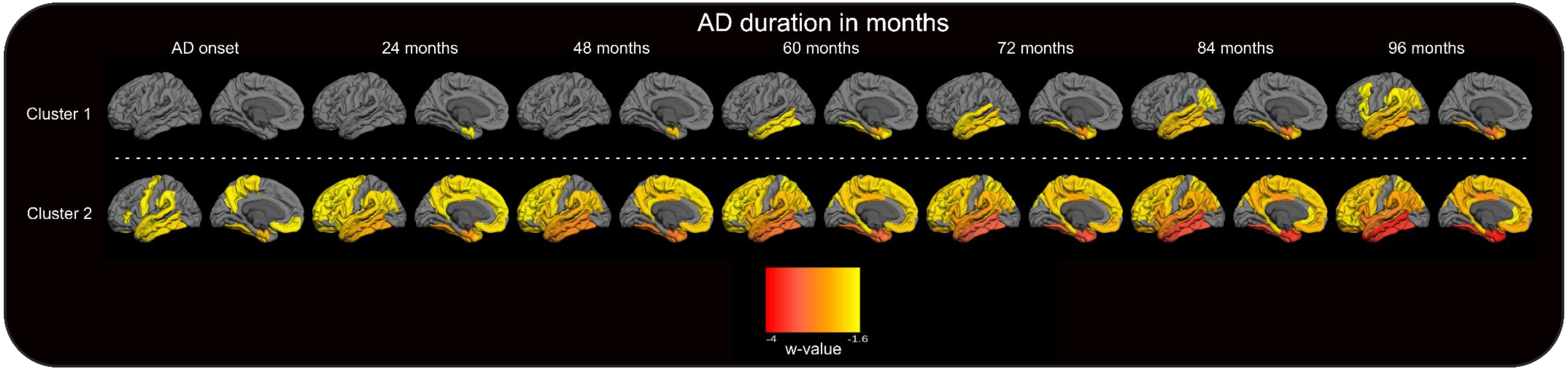

Figure S1. 2-cluster solution atrophy fitted value maps

Atrophy fitted values after the AD onset. Each row represents one cluster of patients with the corresponding pattern of atrophy. The colourscale reflects to AD atrophy compared to a multicohort dataset of $A\beta$ negative cognitively unimpaired (CU) individuals. Data are w-value transformed and therefore colours represent standard deviations bellow the CU group controlled for aging. Fitted values are fixed for intracranial volume and MRI scanner field strength.Yellow and red represent less and more atrophy respectively.

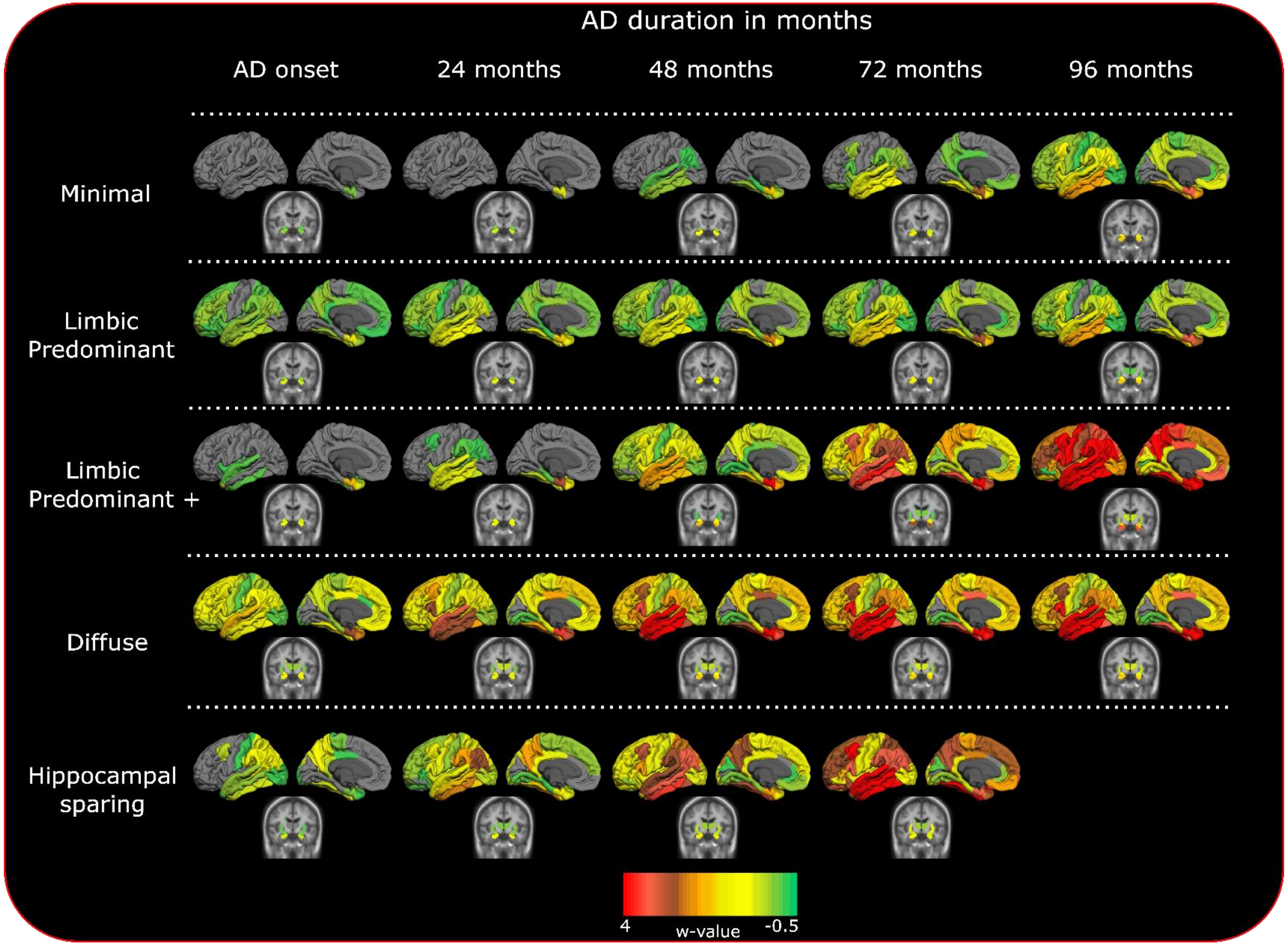

Figure S2. Fitted values for cortical thickness and subcortical volumes for the different longitudinal patterns of atrophy from AD onset. Atrophy fitted values from

clinical AD onset. Each row represents one cluster of patients with the corresponding pattern of atrophy. The color scale illustrates cortical thinning and subcortical volume loss compared to Aβ negative, cognitively unimpaired (CU) individuals. Data are w-value transformed and therefore colors represent standard deviations below the CU group controlled for aging. Fitted values are fixed for intracranial volume and MRI scanner field strength. This figure is an uncorrected (less conservative) version of Figure 2 of the main manuscript (1.6 standard deviations below CU sample, ~95%), but the imaging threshold is set to 0.5 standard deviations below the CU sample normative values that correspond to approximately 20% below the CU populations' mean profile assuming normally distributed atrophy data.

.

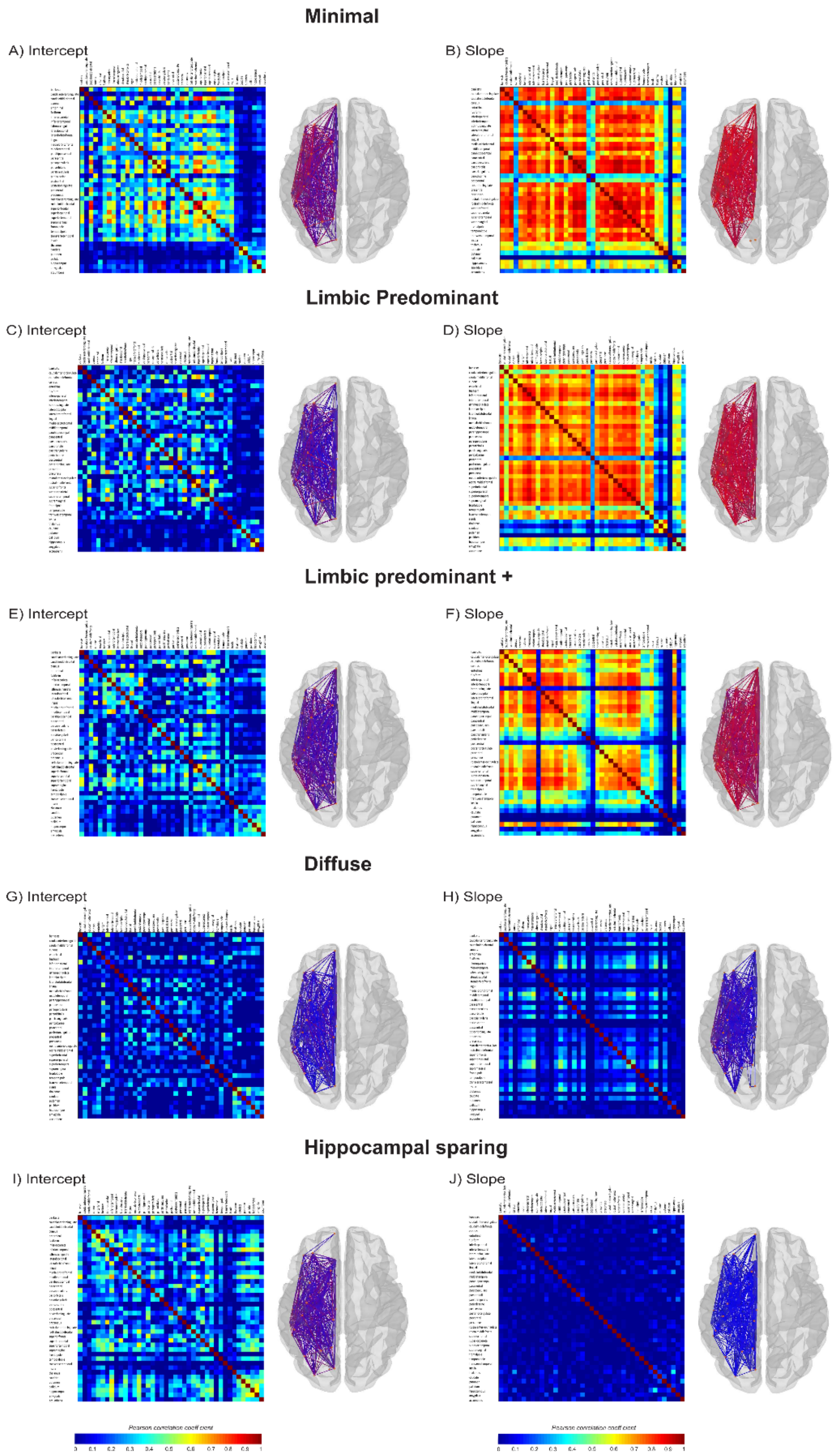

Figure S3. Cluster-specific intercept and slope covariance matrix

The data are presented as correlations instead of variance/covariance in heatmaps form. A, C, E, G, I show cluster intercepts while B, D, F, H, J represent slopes. On the right side of each heatmap we present the topological order the relationship between variables with edges between brain regions coloured according to correlation. The colour scale spans from blue to red with low and high correlations, respectively.

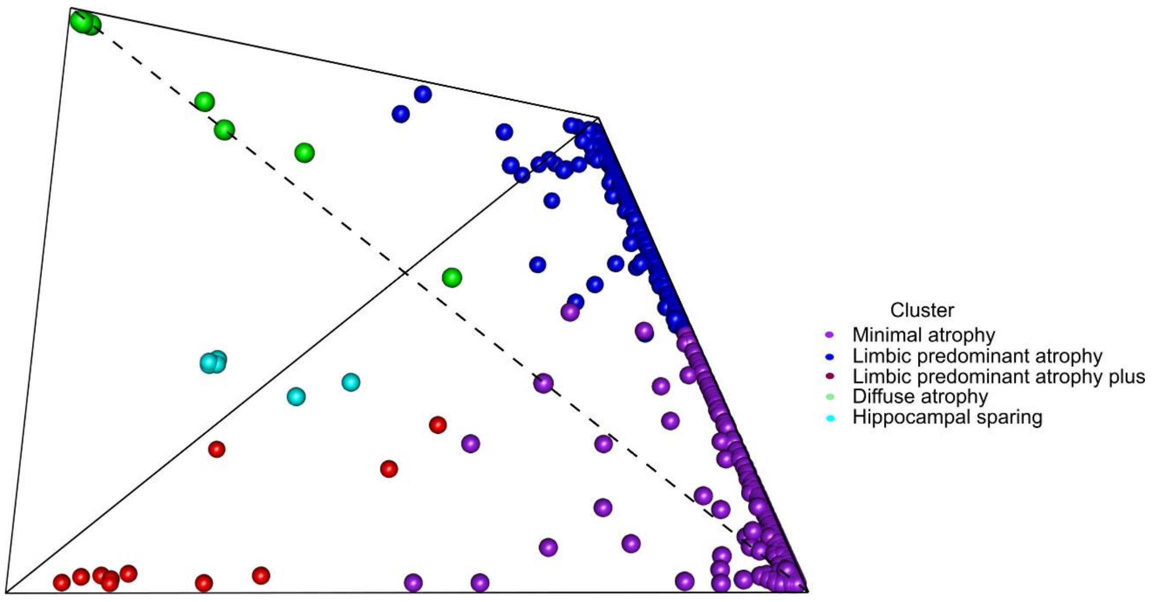

Figure S4. Validation dataset's patient's component probabilities figure.

This figure shows the 1st, 2nd, and 3rd principal component analysis coordinates for the patient cluster probabilities matrix of the discovery dataset. Only four out of the five hundred seventy one principal components were above near zero values. In the figure, each dot represents one patient of the discovery dataset. Any coordinate within the pyramidal plot, represents different patient cluster probability compositions, e.g., closest to the lower right corner, we can observe patients that are certainly clustered at the minimal atrophy cluster of patients. Since only the first three principal components are visualized, the corner of certain classification for the hippocampal sparing cluster of patients is not visualized. The first three principal components are enough to summarize information that separates the hippocampal sparing cluster from the other clusters of patients. Only 5 out of 571 patients are not certainly classified to one of the five clusters (see Table S5). The minimal and limbic predominant atrophy patterns show the greatest similarity (many AD patient probabilities trace dots lie on the borders between the two clusters).

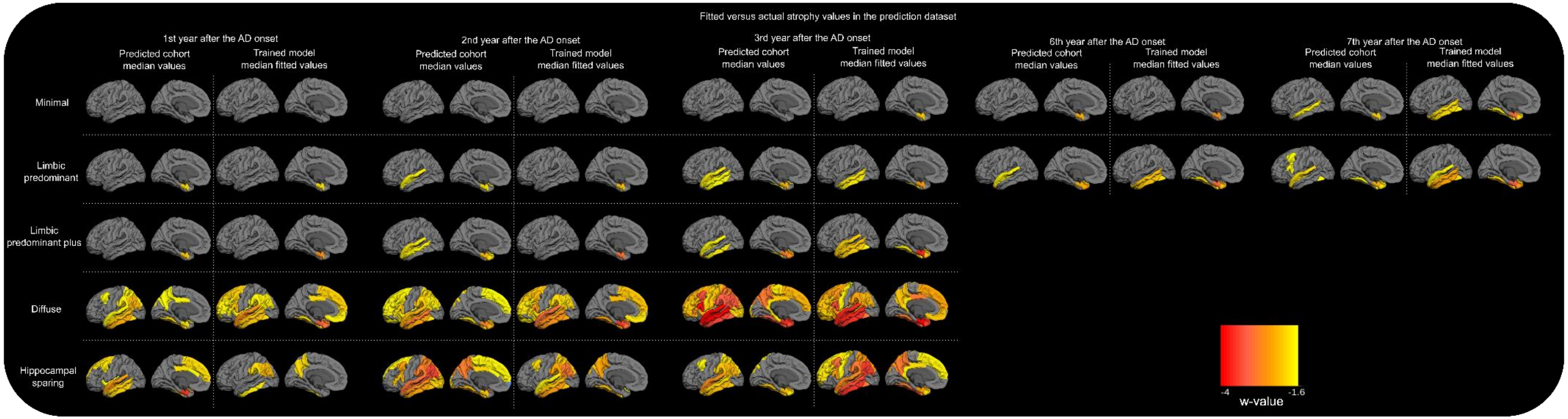

Figure S5. Atrophy fitted values after the AD onset for the trained clustering model versus the new validation dataset data.

For the 1st, 2nd, 3rd, 6th and 7th years after the AD onset, the validation dataset had enough data to provide median atrophy images per cluster. These new observations were classified to each cluster, and median disease duration was calculated. Median atrophy maps (group median atrophy) for the new data of each cluster are presented in the left column of each year's category. Then atrophy fitted values at the median disease duration of each cluster were calculated through the clustering model (right column of each year's category). The colourscale of the cortical maps reflects AD atrophy levels compared to a multicohort dataset of $A\beta$ negative cognitively unimpaired (CU) individuals. Data are w-value transformed and therefore colours represent standard deviations bellow the CU group controlled for aging. Fitted values are fixed for intracranial volume and MRI scanner field strength. Yellow and red represent less and more atrophy respectively.

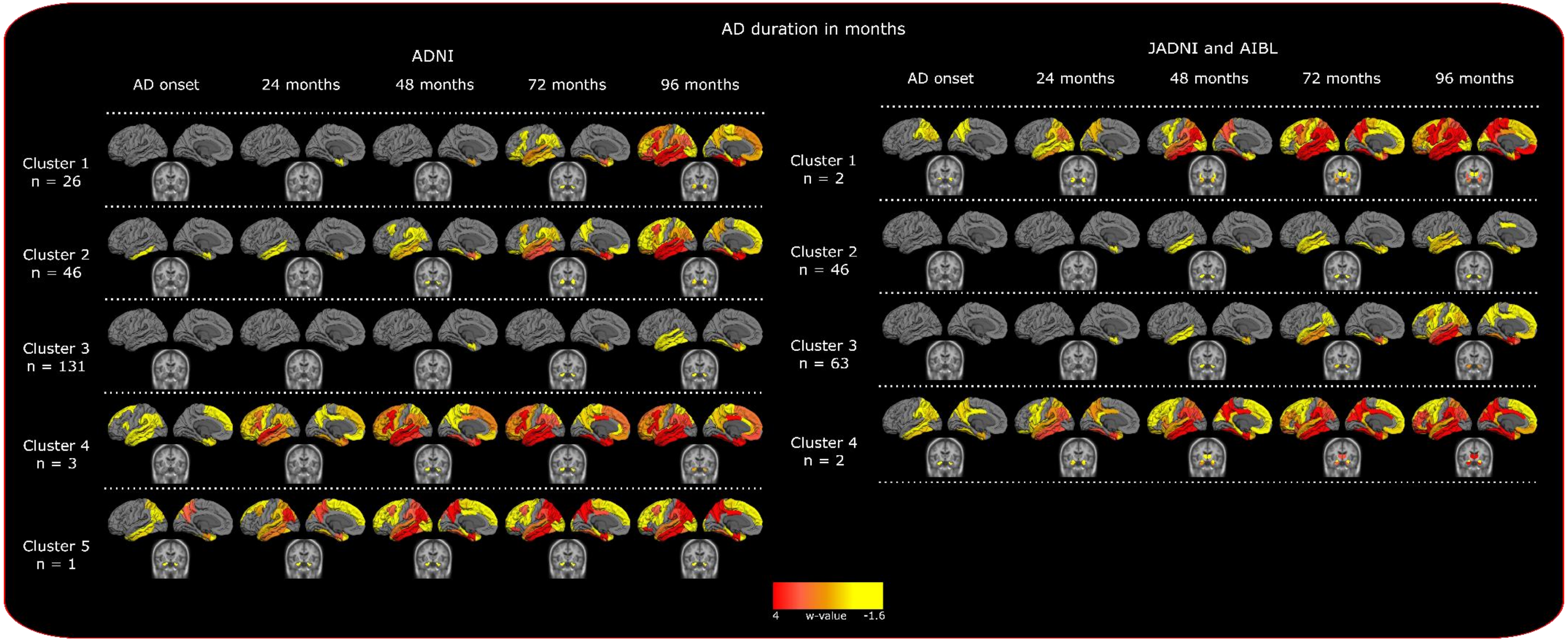

Figure S6. Atrophy fitted values after the AD onset for ADNI (left panel) and J-ADNI/AIBL (right panel).

Each row represents one cluster of patients with the corresponding pattern of atrophy. The colour scale reflects to AD atrophy compared to a multicohort dataset of $A\beta$ negative cognitively unimpaired (CU) individuals. Data are w-value transformed and therefore colours represent standard deviations bellow the CU group controlled for aging. Fitted values are fixed for intracranial volume and MRI scanner field strength. Yellow and red represent less and more atrophy respectively. Imaging threshold is set to -1.6 standard deviations below the CU sample normative values.

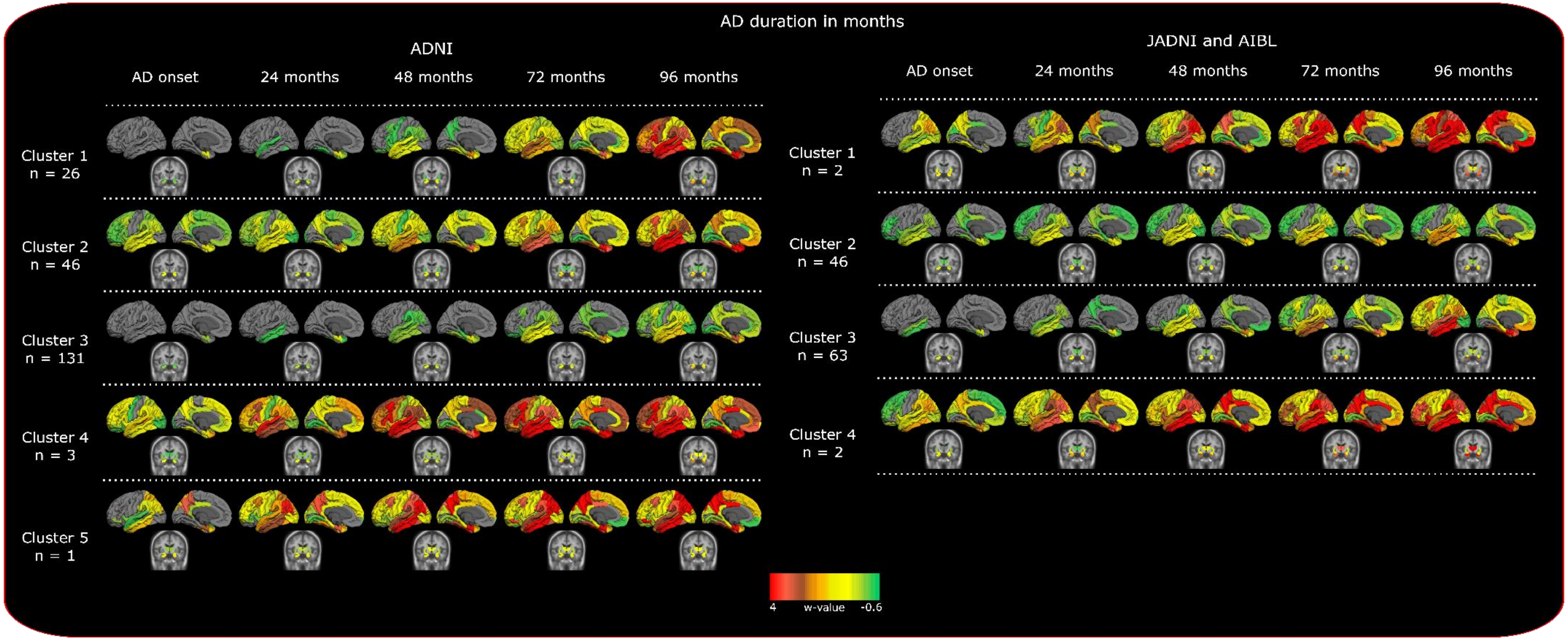

Figure S7. Atrophy fitted values after the AD onset for ADNI (left panel) and J-ADNI/AIBL (right panel).

Each row represents one cluster of patients with the corresponding pattern of atrophy. The colour scale reflects to AD atrophy compared to a multicohort dataset of $A\beta$ negative cognitively unimpaired (CU) individuals. Data are w-value transformed and therefore colours represent standard deviations bellow the CU group controlled for aging. Fitted values are fixed for intracranial volume and MRI scanner field strength. Green and red represent no/much atrophy respectively. Imaging threshold is set to -0.5 standard deviations below the CU sample normative values that correspond to approximately 20% of the CU population assuming normally distributed atrophy data. This figure can be considered as an uncorrected version of the figure S8. The quantitative analysis for the assessment of similarities between the ADNI and J-ADNI/AIBL longitudinal atrophy patterns, showed that ADNI atrophy pattern 1 is more similar to J-ADNI/AIBL pattern 3, ADNI atrophy pattern 2 is more similar to J-ADNI/AIBL pattern 2, ADNI atrophy pattern 3 is more similar to J-ADNI/AIBL pattern 2, ADNI atrophy pattern 4 is more similar to J-ADNI/AIBL pattern 4, and ADNI atrophy pattern 5 is more similar to J-ADNI/AIBL pattern 4. J-ADNI/AIBL pattern 1

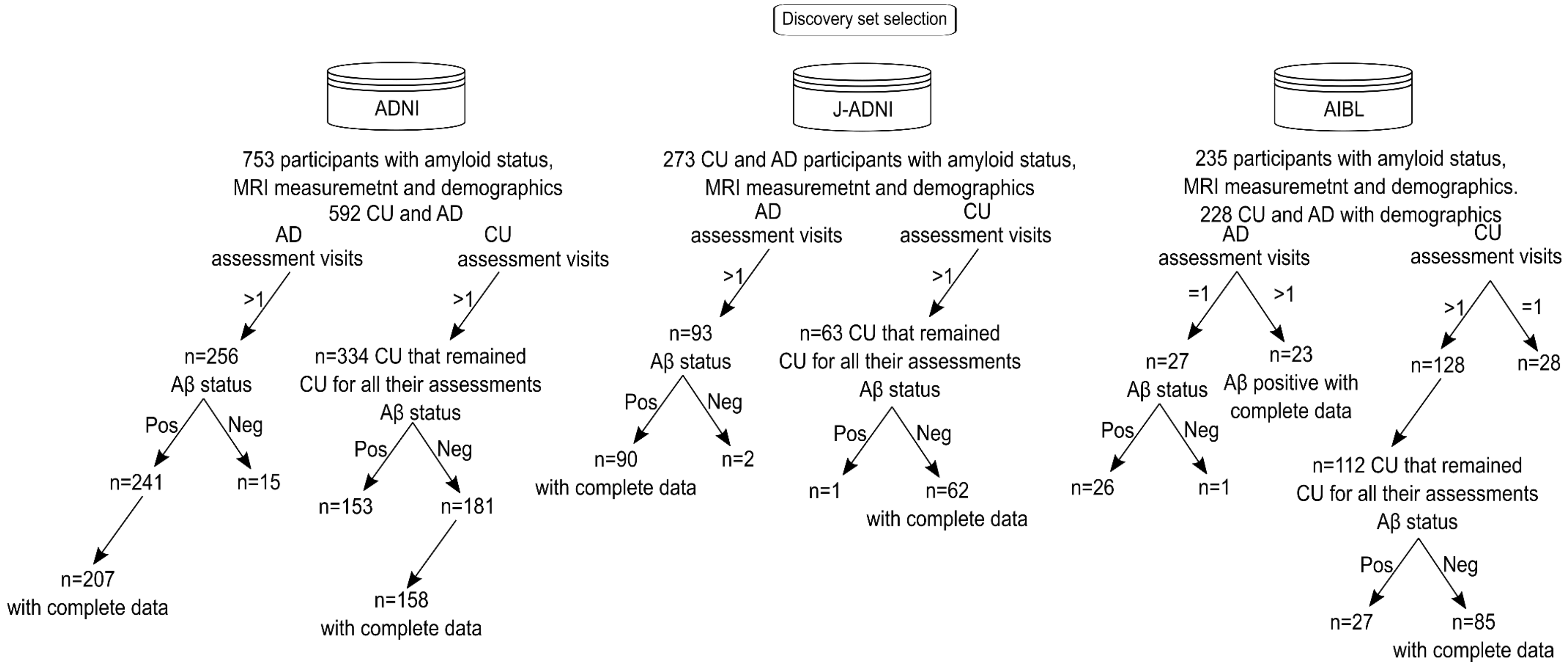

Figure S8. Discovery set participants

In the J-ADNI dataset 117 individuals were excluded because only 69 of them were AD or CU. 28 out of 69 participants were CU, and only 18 remained CU (With no imaging data) for all cognitive assessments while the rest progressed to mild cognitive impairment. 41 out of 69 participants were diagnosed with AD and only 3 had repeated measurements imaging data that did not pass the manual FreeSurfer quality control assessment.

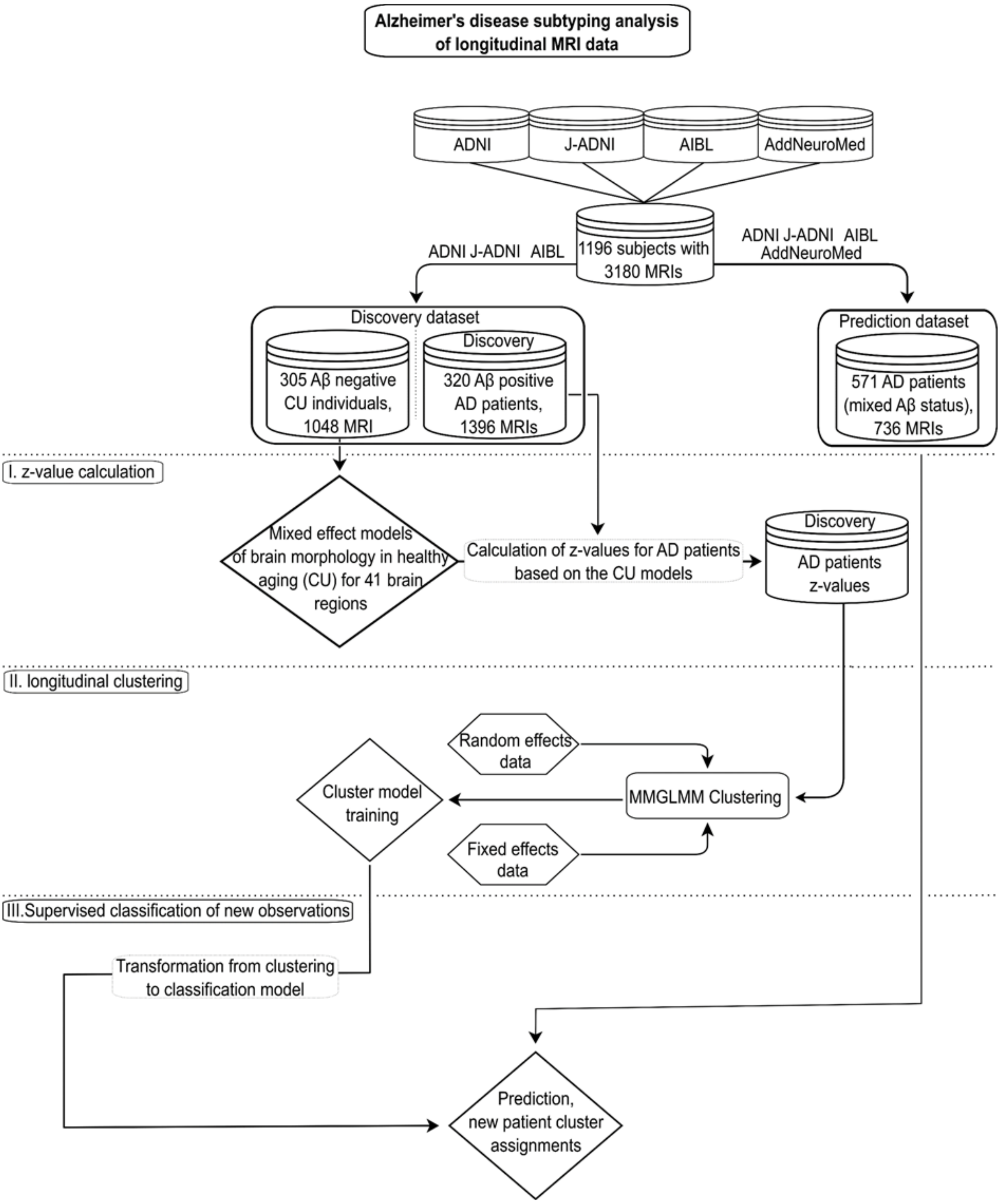

Figure S9. Study flowchart

Supplementary References

1. Komarek, Arnost, and Lenka Komárková. "Clustering for multivariate continuous and discrete longitudinal data." The Annals of Applied Statistics (2013): 177-200.
