# Supplementary material for: Multi-cohort and longitudinal Bayesian clustering study of stage and subtype in Alzheimer’s disease
Source: Nat Commun. 2022 Aug 5;13:4566. doi: 10.1038/s41467-022-32202-6 (PMC9355993; doi:10.1038/s41467-022-32202-6)
Supplement: Supplementary file 3 — Description of Additional Supplementary Files [file 41467_2022_32202_MOESM3_ESM.pdf]

Description of Additional Supplementary Files

File Name: Supplementary Movie 1
Description: Video Abstract

File Name: Supplementary Software 1

Description: Model R Code Archive
