# Supplementary material for: Multi-cohort and longitudinal Bayesian clustering study of stage and subtype in Alzheimer’s disease
Source: Nat Commun. 2022 Aug 5;13:4566. doi: 10.1038/s41467-022-32202-6 (PMC9355993; doi:10.1038/s41467-022-32202-6)
Supplement: Supplementary file 6 — Reporting Summary [file 41467_2022_32202_MOESM6_ESM.pdf]

nature research

Corresponding author(s): Konstantinos Poulakis

Last updated by author(s): May 30, 2022

Reporting Summary

Nature Research wishes to improve the reproducibility of the work that we publish. This form provides structure for consistency and transparency in reporting. For further information on Nature Research policies, see our Editorial Policies and the Editorial Policy Checklist.

Statistics

For all statistical analyses, confirm that the following items are present in the figure legend, table legend, main text, or Methods section.

n/a	Confirmed	
☐	☒	The exact sample size (*n*) for each experimental group/condition, given as a discrete number and unit of measurement
☐	☒	A statement on whether measurements were taken from distinct samples or whether the same sample was measured repeatedly
☐	☒	The statistical test(s) used AND whether they are one- or two-sided<br>*Only common tests should be described solely by name; describe more complex techniques in the Methods section.*
☐	☒	A description of all covariates tested
☐	☒	A description of any assumptions or corrections, such as tests of normality and adjustment for multiple comparisons
☐	☒	A full description of the statistical parameters including central tendency (e.g. means) or other basic estimates (e.g. regression coefficient) AND variation (e.g. standard deviation) or associated estimates of uncertainty (e.g. confidence intervals)
☐	☒	For null hypothesis testing, the test statistic (e.g. *F*, *t*, *r*) with confidence intervals, effect sizes, degrees of freedom and *P* value noted<br>*Give P values as exact values whenever suitable.*
☐	☒	For Bayesian analysis, information on the choice of priors and Markov chain Monte Carlo settings
☐	☒	For hierarchical and complex designs, identification of the appropriate level for tests and full reporting of outcomes
☒	☐	Estimates of effect sizes (e.g. Cohen's *d*, Pearson's *r*), indicating how they were calculated

Our web collection on statistics for biologists contains articles on many of the points above.

Software and code

Policy information about availability of computer code

Data collection	No specific software was used for data collection. Freesurfer 6.0.0 was used for data processing and volumetric/thickness estimations. BRAPH v 1.0.0 for the graph analysis. R 3.6.3 was used for the data analysis including processing, clustering and demographics.
Data analysis	The code for the preprocessing, main analysis, and post processing is provided in separate file and will be added as supplementary material.

For manuscripts utilizing custom algorithms or software that are central to the research but not yet described in published literature, software must be made available to editors and reviewers. We strongly encourage code deposition in a community repository (e.g. GitHub). See the Nature Research guidelines for submitting code & software for further information.

Data

Policy information about availability of data

All manuscripts must include a data availability statement. This statement should provide the following information, where applicable:
- Accession codes, unique identifiers, or web links for publicly available datasets
- A list of figures that have associated raw data
- A description of any restrictions on data availability

The datasets generated and analysed during the current study are not available on their entirety due to individual agreements with the four cohort (ADNI, JADNI, AIBL, AddNeuroMed) committees. The datasets can be acquired after request to the individual cohort repositories. Unique deidentified ids of patients in each cluster and the clustering results and full models outputs can be shared upon reasonable request. TheHiveDB was used for processing of images with Freesurfer 6.0.0.

Field-specific reporting

Please select the one below that is the best fit for your research. If you are not sure, read the appropriate sections before making your selection.

- [x] Life sciences
- [ ] Behavioural & social sciences
- [ ] Ecological, evolutionary & environmental sciences

For a reference copy of the document with all sections, see nature.com/documents/nr-reporting-summary-flat.pdf

Life sciences study design

All studies must disclose on these points even when the disclosure is negative.

Sample size	For AD the sample size in the discovery and validation datasets was decided in terms of information maximization in the discovery dataset. We focused on including as many abeta positive AD patietns with more than one MRIs available as possible from the three cohorts (ADNI, J-ADNI, AIBL). The validation dataset included all patients from the four cohorts (previous plus AddNeuromed), that had at least one MRI available.<br>For cognitively unimpaired individuals, abeta negative individuals that had at least two MRIs available and were cognitively unimpaired during all their future clinical follow ups were included.
Data exclusions	Only data that failed the manual quality control (coronal slice inspection, slice by slice for each image) of freesurfer were excluded from further analysis. More specifically, images for which Freesurfer 6.0.0 parcellation was wrong (delineation included), were excluded from further analysis.
Replication	The model was evaluated in the evaluation cohort (see methods). The model parameters were used to train a classifier which was used to predict the cluster allocation of each new patient's MRI. Then, fitted values of the clustering model were contracted to the actual MRI images to inspect whether the classification failed or not to assign each image to the closest cluster's fitted value (see figure 3 of the main manuscript).<br>The model was applied separately to the ADNI and J-ADNI/AIBL cohorts to assess the similarities of patterns and performance of the clustering model in the separate datasets (see supplementary file 1).
Randomization	Participants were allocated in the cognitively unimpaired or Alezheimer's disease dementia groups with the help of clinical diagnosis.
Blinding	Cognitively unimpaired individuals were separated from the AD patients by the investigators based on their diagnostic labels.<br>The main analysis is unsupervised. Thus, the investigators were blinded to cluster allocation. The cluster allocation was a production of probabilistic clustering.

Reporting for specific materials, systems and methods

We require information from authors about some types of materials, experimental systems and methods used in many studies. Here, indicate whether each material, system or method listed is relevant to your study. If you are not sure if a list item applies to your research, read the appropriate section before selecting a response.

Materials & experimental systems

n/a	Involved in the study	
x		Antibodies
x		Eukaryotic cell lines
x		Palaeontology and archaeology
x		Animals and other organisms
	x	Human research participants
	x	Clinical data
x		Dual use research of concern

Methods

n/a	Involved in the study	
x		ChIP-seq
x		Flow cytometry
	x	MRI-based neuroimaging

Human research participants

Policy information about studies involving human research participants

Population characteristics	Alzheimer's disease and cognitively unimpaired individuals (CU). CU individuals have to be CU in past, present and future diagnosis (our study is retrospective). 55-90 years old, both sexes, modified hachinski score less or equal to 4, CDR depending on diagnosis (0 for CU, 0.5 or 1 for AD), MMSE depending on diagnossi and variable between cohorts, Geriatric Depression Scale score of <6.
Recruitment	Participants were recruited from the participating clinics in each cohort depending on their diagnosis. The cohorts included in the study can be described as a non-randomized natural history non-treatment study. Potential biases can rise from the longitudinal nature of the participant selection. That is, only participants with more than one MRIs were included in the discovery dataset modeling. Therefore, AD patients that declined rapidly and were not able to participate for a new MRI examination were excluded from the cluster formation. However, this small subset was included in the evaluation of the model and their data were also analyzed for model evaluation purposes.
Ethics oversight	This study was approved by the Institutional Review Boards of all of the participating institutions (ADNI, J-ADNI, AddNeuroMed, AIBL). Informed written consent was obtained from all participants at each site. All subjects were required to provide informed consent as compatible with the local sites (Institutional Review Board regulations).

Note that full information on the approval of the study protocol must also be provided in the manuscript.

Clinical data

Policy information about clinical studies

All manuscripts should comply with the ICMJEguidelines for publication of clinical research and a completedCONSORT checklist must be included with all submissions.

Clinical trial registration	*Provide the trial registration number from ClinicalTrials.gov or an equivalent agency.*
Study protocol	*Note where the full trial protocol can be accessed OR if not available, explain why.*
Data collection	*Describe the settings and locales of data collection, noting the time periods of recruitment and data collection.*
Outcomes	*Describe how you pre-defined primary and secondary outcome measures and how you assessed these measures.*

Magnetic resonance imaging

Experimental design

Design type	structural MRI
Design specifications	*Specify the number of blocks, trials or experimental units per session and/or subject, and specify the length of each trial or block (if trials are blocked) and interval between trials.*
Behavioral performance measures	*State number and/or type of variables recorded (e.g. correct button press, response time) and what statistics were used to establish that the subjects were performing the task as expected (e.g. mean, range, and/or standard deviation across subjects).*

Acquisition

Imaging type(s)	structural
Field strength	1.5 and 3T
Sequence & imaging parameters	MPRAGE sequences and ultra fast MPRAGE sequences. Variable in the different cohorts.
Area of acquisition	Whole brain

Diffusion MRI ☐ Used ☒ Not used

Preprocessing

Preprocessing software	Freesurfer 6.0.0
Normalization	Freesurfer default pipeline
Normalization template	Freesurfer default pipeline
Noise and artifact removal	Freesurfer default pipeline

Volume censoring | Freesurfer default pipeline

Statistical modeling & inference

Model type and settings: Multivariate longitudinal clustering with fixed (population), random (time scale) effects, and gaussian mixture modelling to identify clusters

Effect(s) tested: *Define precise effect in terms of the task or stimulus conditions instead of psychological concepts and indicate whether ANOVA or factorial designs were used.*

Specify type of analysis: ☐ Whole brain ☒ ROI-based ☐ Both

Anatomical location(s): Desikan atlas, hemispheric data averaged

Statistic type for inference (See Eklund et al. 2016): *Specify voxel-wise or cluster-wise and report all relevant parameters for cluster-wise methods.*

Correction: only one model is considered (where all parameters are considered simultaneously using full conditional distributions) and therefore there are no multiple corrections in the main analysis

Models & analysis

n/a	Involved in the study
☐	☐ Functional and/or effective connectivity
☐	☐ Graph analysis
☐	☒ Multivariate modeling or predictive analysis

Functional and/or effective connectivity: *Report the measures of dependence used and the model details (e.g. Pearson correlation, partial correlation, mutual information).*

Graph analysis: *Report the dependent variable and connectivity measure, specifying weighted graph or binarized graph, subject- or group-level, and the global and/or node summaries used (e.g. clustering coefficient, efficiency, etc.).*

Multivariate modeling and predictive analysis: preprocessing: univariate (one response) mixed effect models were trained on the cognitvely unimpaired individuals for each ROI (41 ROIs). The fixed effect was age and radom effect was subject id and cohort. Individuals had up to 11 MRI follow ups. AD patients z values were calculated based on fitted values of the univariate CU based mixed effect models to account for the aging effect in grey matter and the cohort effect.

Main analysis: As described above (Statistical modeling & inference), multivariate clustering modelling was applied to the AD discovery dataset.

Post clustering: Multivariate longitudinal mixed effect classifier (mixture model) was trained based on the clustering model of the main analysis. The evaluation data that are independent observations (new unseen AD patietns) were classified into one of the classes (clusters). Then the atrophy patterns of the raw data were compared to the clustering model's (main analysis) fitted values to assess the ability of the model to correctly assign patterns of atrophy after the AD onset to one of the discovered clusters.
